# Steering Wheel Aftermarket Rhinestone Emblem Projectile Injury in a Motor Vehicle Collision: A Case Report

**DOI:** 10.7759/cureus.56626

**Published:** 2024-03-21

**Authors:** Benjamin P Nguyen, Amanda J Khouri, Ferdinand Perez-Rodriguez, Vincent E Kirkpatrick, Sebastiano Cassaro

**Affiliations:** 1 Department of Surgery, Kaweah Health Medical Center, Visalia, USA

**Keywords:** airbag deployment, airbag-associated injury, motor vehicle collison, rhinestone emblem, aftermarket emblem, steering wheel

## Abstract

Motor vehicle collisions are a leading cause of morbidity and mortality within the United States. Safety devices such as seatbelts and airbags have significantly reduced mortality rates for drivers. Some drivers personalize their vehicles with inexpensive items that may cause significant injury during vehicular collisions. We present a case of a face and neck penetrating injury from a metal aftermarket rhinestone emblem attached to the steering wheel during a motor vehicle collision. A 43-year-old female was involved in an accident where the airbag had deployed and projected two pieces of the metal emblem at her face and neck. The fragment in the neck was removed at the bedside, while that in the face required removal in the operating room. She recovered well postoperatively and was discharged the same day. This case highlights the potential for the dangers caused by aftermarket rhinestone emblems on steering wheels. We recommend that increased national advisories and legislative actions be considered to limit the use of these emblems.

## Introduction

In 2021, there were 6.1 million motor vehicle collisions within the United States resulting in approximately 40,000 fatalities and 1.7 million injuries [1]. Safety features such as seatbelts and airbags have significantly decreased mortality for drivers [2,3]. However, injuries from airbag deployment including head, neck, torso, upper limb, and lower limb injuries can occur [4-6].

As cars become personal household items, consumers have a variety of ways to customize their vehicles. Steering wheel emblem rhinestone decals are inexpensive and are gaining popularity. These emblems are made of a plastic or metal plate and attached with adhesive to the recesses within the car logo. Though seemingly benign, this rhinestone “bling” can cause serious injury.

The National Highway Traffic Safety Administration (NHTSA) recommends against any aftermarket decals and documented a case where one driver lost sight in one eye when an aftermarket emblem covered with rhinestones hit the driver in the face [7]. Here, we present a case of a 43-year-old woman who was involved in a motor vehicle collision and evaluated as a trauma patient. She was found to have two long narrow metallic objects, originally part of one such emblem, embedded in the anterior soft tissue of the neck and within the right cheek, respectively. To our knowledge, this is the first case report to discuss a penetrating injury from an aftermarket steering wheel rhinestone emblem.

## Case presentation

**Figure 1:**
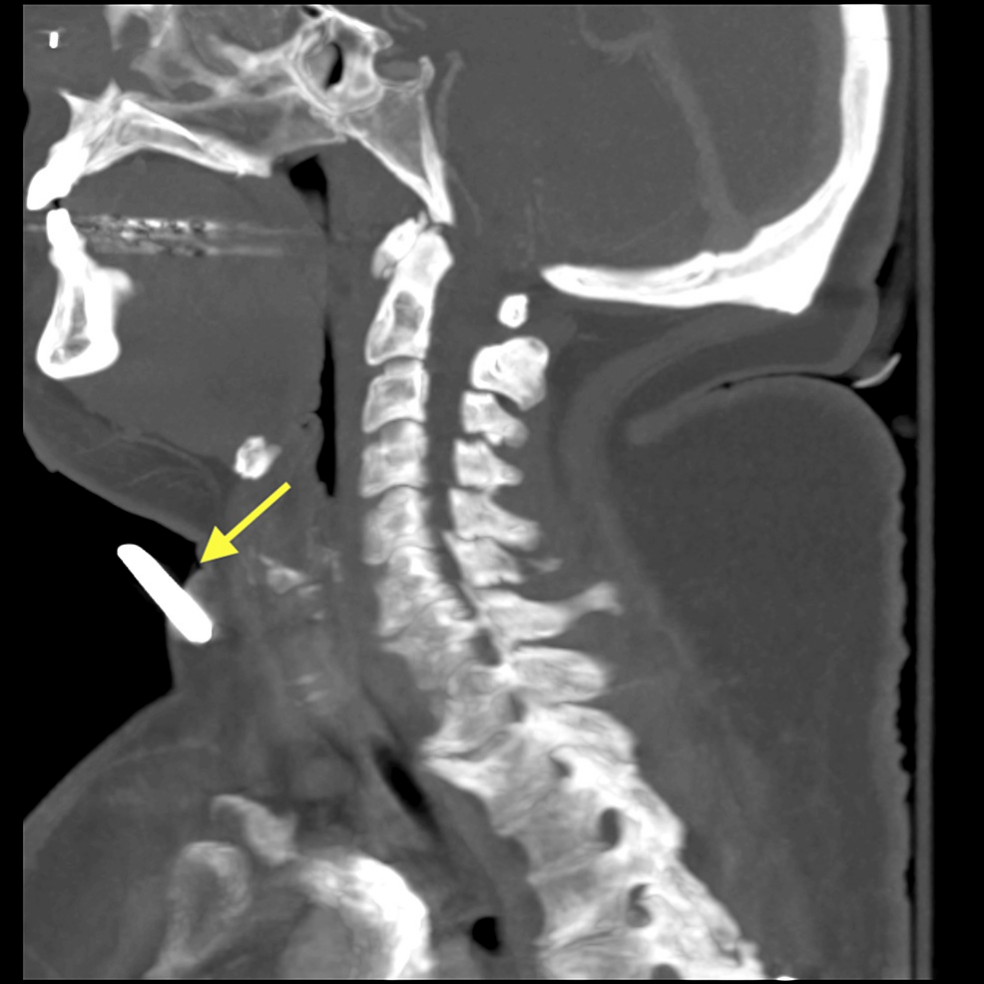
CTA of the neck showing a metallic object penetrating the anterior neck soft tissue CTA: computed tomography angiography

**Figure 2:**
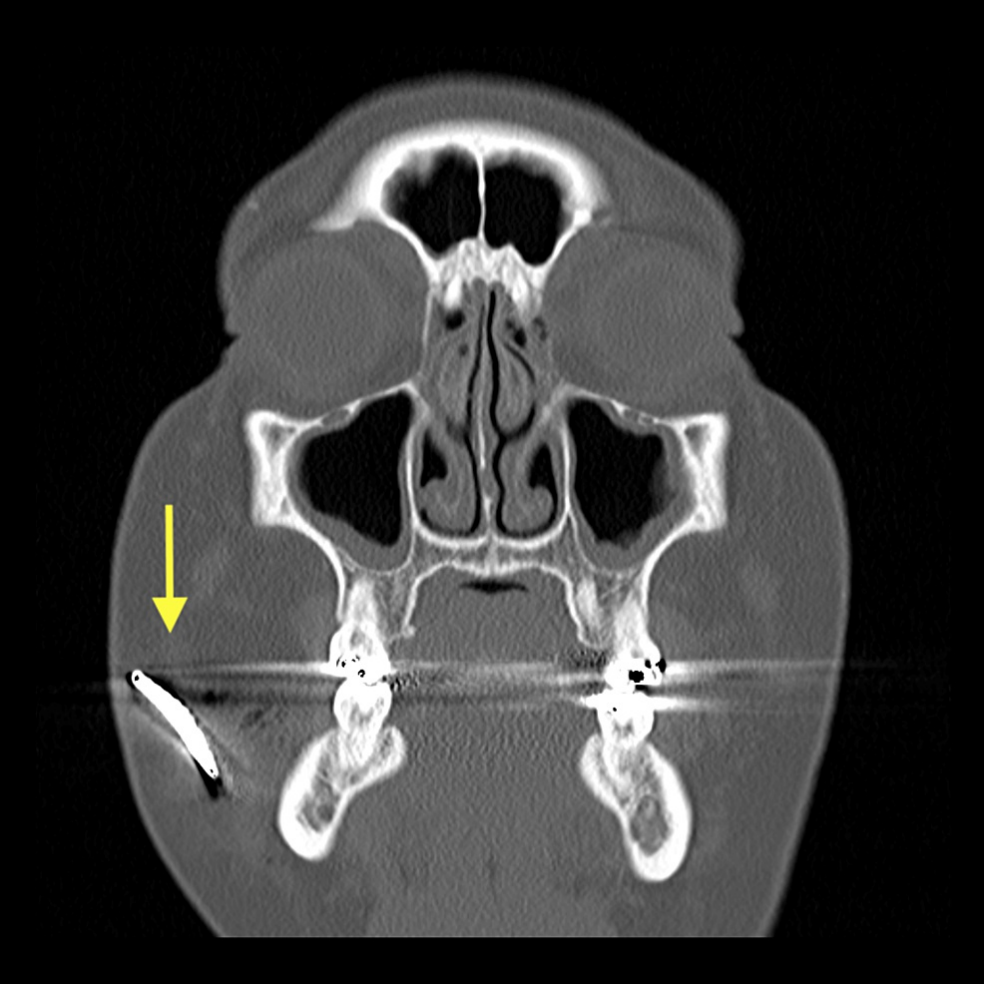
CT maxillofacial scan showing a metallic object embedded in the subcutaneous soft tissues overlying the right maxilla in the coronal view

**Figure 3:**
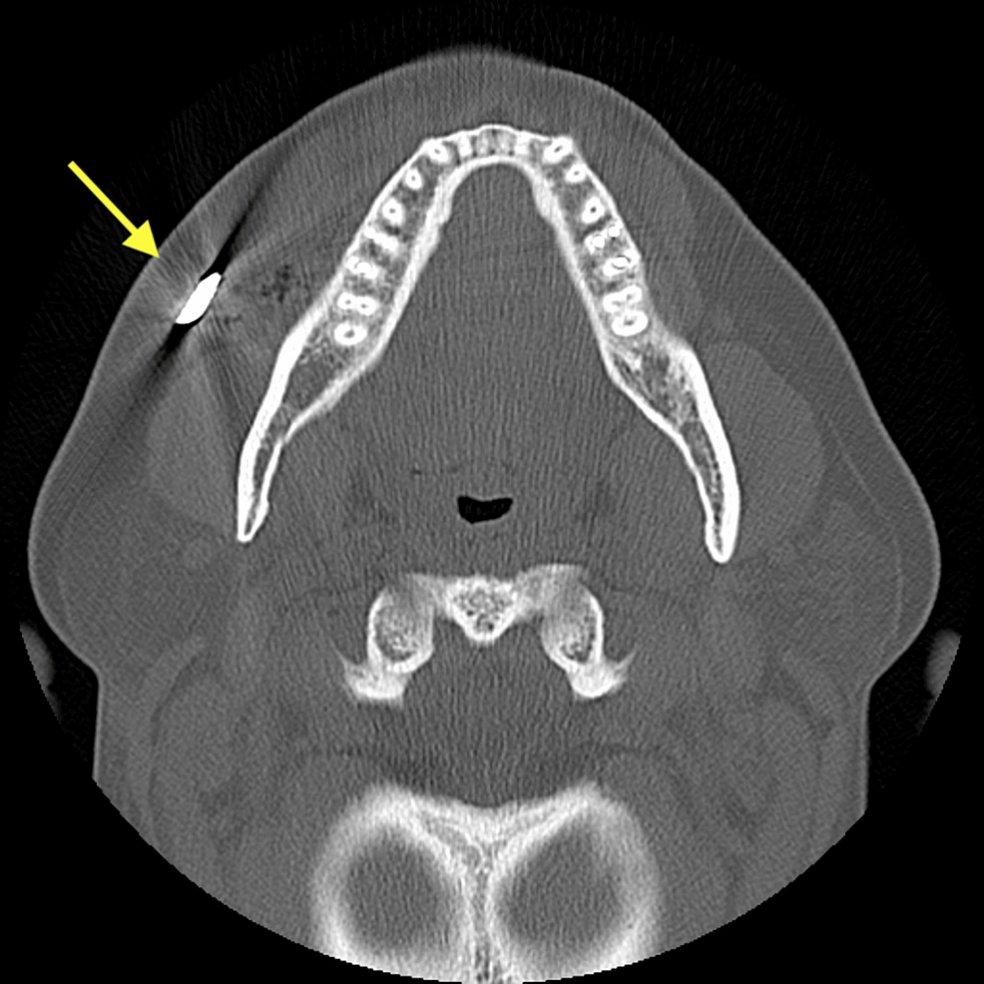
CT maxillofacial scan showing a metallic object embedded in the subcutaneous soft tissues overlying the right maxilla in the axial view

A 43-year-old woman was transported by ambulance to the trauma center for evaluation after a motor vehicle collision. Emergency medical services reported a six-inch intrusion in the driver's side. Her extrication required the assistance of a hydraulic rescue cutter. She was a restrained driver traveling at 25 miles per hour when her car hit the side of another car. Her airbags deployed. She denied loss of consciousness. Vital signs were normal. Laboratory work was unremarkable. She was alert and oriented with a Glasgow Coma Scale score of 15. Her trauma survey was notable for an object within the subcutaneous tissue on the anterior neck, a palpable foreign object in the right cheek with surrounding ecchymosis, two small penetrating wounds in the lower chin, a left lower abdominal wall hematoma, and an abrasion on her left forearm with swelling. A computed tomography angiography (CTA) neck scan was performed revealing a 1.3 cm metallic object penetrating the anterior neck soft tissue (Figure 1) and a CT maxillofacial scan was performed showing a 3 cm metallic object embedded in the subcutaneous soft tissues overlying the right maxilla (Figures 2, 3).

**Figure 4:**
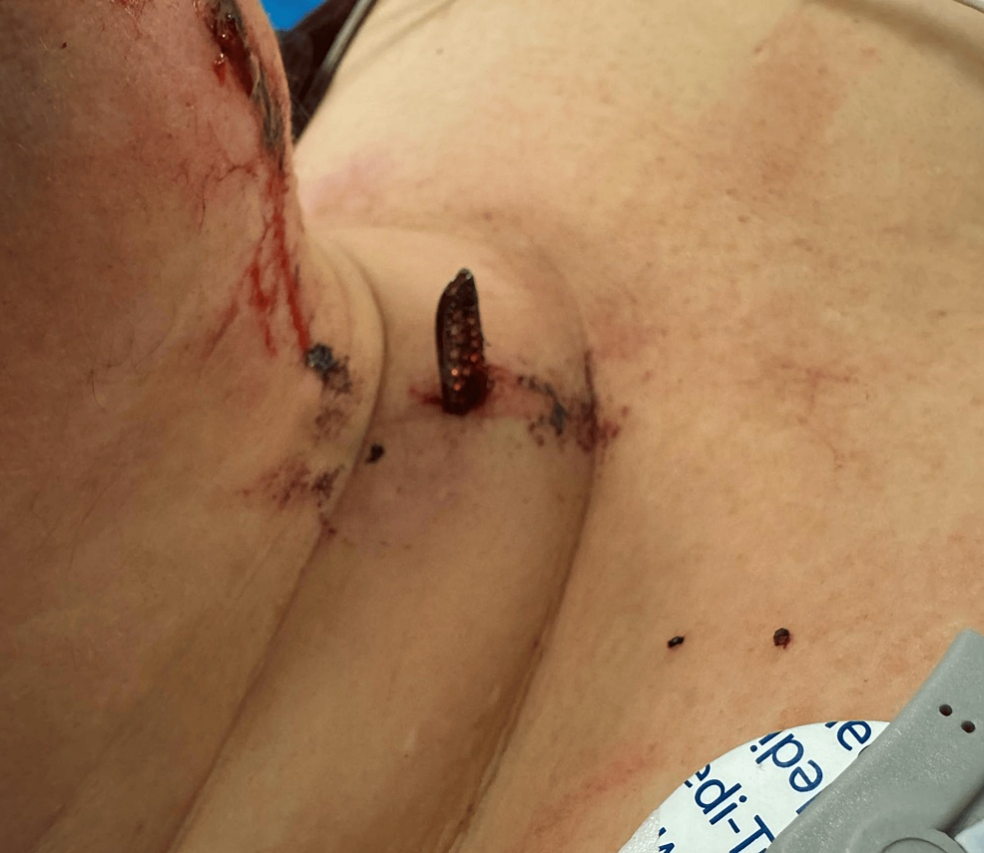
Metallic aftermarket steering wheel rhinestone emblem penetrating the anterior neck soft tissue

**Figure 5:**
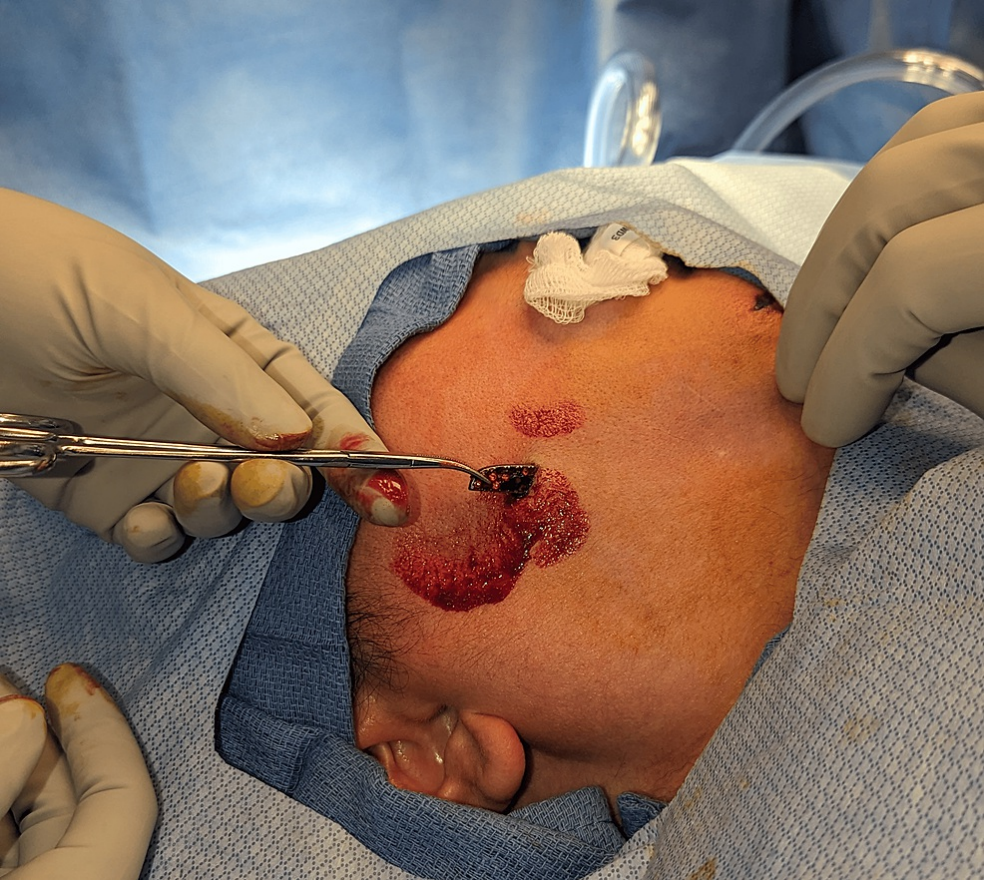
Removal of metallic aftermarket steering wheel rhinestone emblem embedded in the subcutaneous soft tissues overlying the right maxilla

**Figure 6:**
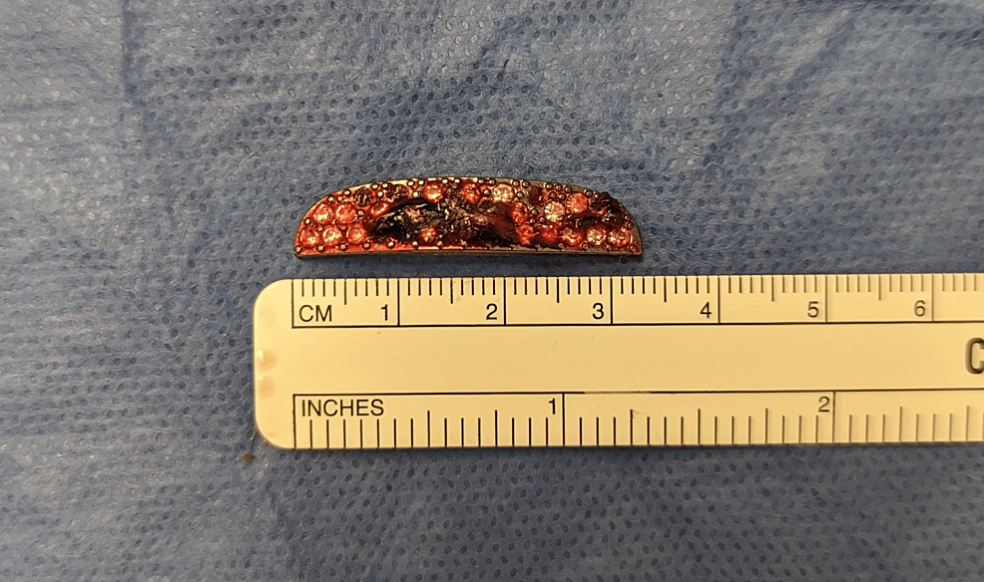
The 3 cm piece of metallic aftermarket steering wheel rhinestone emblem

The 1.3 cm object was removed with traction at bedside without complications (Figure 4). Given the depth of the second foreign object, the patient was taken to the operating room for its removal. In the operating room, a small incision over the tip of the object was made. Using a hemostat, the object was delivered through the incision (Figures 5, 6). The incision was copiously irrigated and closed with interrupted 6-0 polypropylene sutures. The patient was discharged the same day with a five-day course of cephalexin and was instructed to follow up as an outpatient in five days for suture removal.

## Discussion

A retrospective review of airbag injuries by the NHTSA from 1980 to 1994 showed that 96% of all airbag injuries were minor including abrasions, contusions, and lacerations [8]. The most commonly injured areas were the face, wrist, forearm, and chest. In contrast, there have been isolated reports of more serious injuries like decapitation, cervical spine injuries, retinal detachment, cardiac rupture, and heart valve injuries [6]. The injuries described are caused typically by the blunt impact of the airbag facing the front of the driver.

Loose objects inside cars can be a cause of serious injury or death. A survey of 25 cars and drivers revealed that an average of 4.3 potentially dangerous loose everyday objects are inside a vehicle cabin [9]. Possibly due to being uncommon, penetrating injuries in a motor vehicle collision have not been well described in the literature.

Airbags fully expand within 50 milliseconds of the impact [10]. An aftermarket rhinestone emblem is a loose item secured by its adhesive back to the center panel covering the compartment in the hub of the steering wheel that houses the airbag. During the explosive airbag deployment, the fragments of the emblem can become dislodged and act as a high-velocity shrapnel toward the front of the driver with the potential to cause devastating injuries [6,8].

In our patient, one of the emblem pieces penetrated the skin in the lower chin and traveled within the deep soft tissues of the cheek lodging under the skin of the mid cheek. Entering at a different angle, the metal projectiles could have penetrated major vascular structures in the neck or the eye. Many drivers may not be aware of the dangers if their airbags are activated in case of a collision. These emblems are very popular and widely available due to their low cost and aesthetic appeal.

## Conclusions

Motor vehicle collisions are common occurrences and airbags frequently deploy. We presented a case of a steering wheel aftermarket rhinestone emblem being projected by the activation of an airbag into the subcutaneous tissues of a driver’s neck and cheek during a motor vehicle collision. This occurrence highlights the potential of the dangers caused by these “bling” emblems. We recommend more widespread advisories and consideration of legislative action limiting the sales or use of these decorative pieces. Swift action may prevent extensive harm to drivers nationally and worldwide.
